# Doppler-Assessed Ureteric Jet Frequency: A Valuable Predictor of Ureteric Obstruction

**DOI:** 10.7759/cureus.18290

**Published:** 2021-09-26

**Authors:** Waqar Hassan, Imran Sharif, Salman El Khalid, Kausar Ellahibux, Silmi Sultan, Asma Waqar, Agha Zohaib, Fakhir Yousuf

**Affiliations:** 1 Urology, The Kidney Centre Postgraduate Training Institute, Karachi, PAK; 2 Medicine, Karachi Grammar School, Karachi, PAK; 3 Urogynecology, Chandka Medical College, Karachi, PAK

**Keywords:** pregnancy, ureteric stone, ureteric jet frequency, color doppler ultrasonography, hydronephrosis

## Abstract

Objectives: To compare ureterovesical jet frequency in non-obstructed versus obstructed ureter secondary to ureteric stone using ultrasonography in patients presenting with ureteral stones.

Study design: Cross-sectional prospective study.

Place of study and duration: Urology Department, The Kidney Centre Post Graduate Training Institute from May 16 to November 15, 2019.

Methods: This study included 97 patients having presented in the emergency department with acute renal colic and were diagnosed as having ureteral stones on a non-contrast-enhanced computed tomography (NCCT). The ureteric jet frequency was measured by Doppler ultrasonography by our radiologist with the Hitachi Aloka F-37 ultrasound machine after they underwent CT. Patients were asked to drink 750-1000 ml of liquids 15-20 minutes before their ultrasonographic examination of both kidneys, ureters, and urinary bladder. The kidney size (length and width) and presence/absence of hydronephrosis were evaluated by grayscale ultrasound. Then, with the help of color Doppler ultrasonography, the frequency of the ureteric jet was recorded.

Results: The patient's mean age was 46.66 ± 3.21 years ranging from 37 to 56 years. There were 58 (59.8%) male and 39 (40.2%) female cases. The mean cumulative stone size was 9.77 ± 2.65 mm. According to stone location, 44 (45.4%) cases had upper ureteric, 24 (24.7%) cases had mid ureteric, and 29 (29.9%) cases had lower ureteric stone. The mean obstructive side jet frequency was 0.70/min ± 0.49, and the non-obstructive side jet frequency was 2.89/min ± 1.29 (P < 0.05).

Conclusions: The mean obstructive side jet frequency was 0.70 ± 0.49/min, which, if we compare to the non-obstructed normal ureter, is significantly less. Hence, color Doppler ultrasonography can be helpful to patients who were previously diagnosed with ureteral stones on NCCT to see if their stone has passed. This can be a very cost-effective modality especially in resource-poor countries where repeat CT can be very expensive. The results from this study can also be used in a specific population (i.e., pregnancy) where the use of imaging modalities that involve ionizing radiation is prohibited.

## Introduction

Urinary tract stone is fairly common in the general population with a prevalence of 12% among which around 2.3% encounter renal colic in their lifetime [1,2]. Males are more predominantly affected by the urinary tract stone than females, with the male to female ratio ranging from 1.3 to 5 in some of the studies conducted in Asia [3]. Ureteral stones are fairly common of all urinary tract stones, with ureteral stones at around 20% [4,5]. Historically, for diagnosis of ureteral stone, plain radiograph or intravenous pyelography was carried out, which had several disadvantages from the risk of radiation exposure to low sensitivity at detecting ureteric stone. Ultrasound kidney, ureter, and bladder (KUB) and non-contrast-enhanced computed tomography (NCCT), therefore, remain the most used investigations for diagnosis of ureteric stone. NCCT is far more superior to ultrasonography in detecting ureteric stones, but because of ionizing radiation and lack of availability in many centers, ultrasonography became a valuable alternative tool for the evaluation of patients with ureteric stones [6,7]. In specific conditions such as pregnancy, there exists a concern regarding the use of ionizing radiation that has potential teratogenic effects on the fetus. So using ultrasonography, radiation exposure can be minimized in such patients. It is for this reason that several guidelines including the European Association of Urology (EAU), American Association of Urology (AUA), and American College of Radiology (ACR) have recommended ultrasonography as the first-line modality for acute renal colic in young or pregnant females [8-10].

When the urine flows from the ureter into the bladder, the ultrasound shows it as a urine jet coming out of the ureter. It is speculated that the number of urinary jets coming out of the ureter can vary significantly in obstructed ureter compared to the non-obstructed ureter. The information from the number of urine jets can help improve the diagnostic accuracy of ultrasonography in detecting ureteric stones. There are some studies by few investigators, which help to find out the number of ureteric jets in the normal and obstructed ureters using ultrasonography [11-17]. In a study conducted by Jandaghi et al. [18], the frequency of ureteric jet on the obstructed side was reported as 0.59 ± 0.90 jet/min in patients presenting with ureteric stones [15,18-21].

This study aimed to assess the ureteric jet frequency of the non-obstructed and obstructed ureters using Doppler ultrasonography. The results from this data can help us determine if the stone has passed based on the findings of a ureteric jet. By doing so, not only patients are exposed to less ionizing radiation from computed tomography (CT) scans, but it can also be cost-effective.

## Materials and methods

This cross-sectional prospective study was conducted at the Department of Urology and Radiology of The Kidney Centre Post Graduate Training Institute, Karachi, Pakistan. The duration of the study was from May 16 to November 15, 2019. All the patients who presented to our emergency department or who were in an outpatient clinic with acute renal colic who underwent NCCT, had confirmed ureteric stone on NCCT, and met the inclusion criteria were selected for this study after their informed consent. Patients with bilateral ureteric stones were excluded from the study. Ultrasonography was done by a single consultant radiologist using the Hitachi Aloka F-37 ultrasound machine after confirmation of ureteric stone on NCCT. Ultrasonography was complimentary imaging provided to the patients who were enrolled in the study. Patients were asked to drink 750-1000 ml of liquid 15 to 20 minutes before their ultrasonographic examination of kidneys, ureters, and urinary bladder. The kidney size (length and width) and presence/absence of hydronephrosis were recorded using ultrasonography. Doppler ultrasound was used to assess the frequency of ureteric jets/min for 10 minutes arising from each ureter, obstructed and non-obstructed. The information was then recorded on the principal investigator's laptop in digital form.

The data was then analyzed with Statistical Package for the Social Sciences (SPSS) version 21 (IBM Corp., Armonk, NY). Continuous variables such as age, creatinine, weight, kidney size, stone size, and ureteric jet frequency/min on the obstructed and unobstructed sides were presented as mean and standard deviation. Frequency and percentages were calculated for gender, side of kidney, presence or absence of hydronephrosis, and stone location. The effects of modifiers such as age, kidney size and site, presence or absence of hydronephrosis, stone size, stone’s location in the ureter, and gender were addressed through stratification. Post-stratification, an independent t-test was applied. P-value < 0.05 was considered significant.

## Results

The mean age of patients was 46.66 ± 3.21 years with a minimum of 37 years and a maximum of 56 years. A total of 32 (33%) cases were 30-45-years old and 65 (67%) of the cases were 46-60-years old. There were 58 (59.8%) male and 39 (40.2%) female cases. The mean creatinine levels were 1.03 ± 0.31 mg/dl with minimum and maximum creatinine values as 0.50 and 1.50mg/dl, respectively. The mean length of the right and left kidneys was 116.27 ± 2.33 mm and 115.96 ± 2.51 mm, respectively, while the width of the right and left kidneys was 25.7 ± 2.5 mm and 21.5 ± 26 mm, respectively (Table 1).

**Table 1:** Baseline demographics and clinical characteristics of the patients

Variables	Values
Sex	
Male	58 (59.8%)
Female	39 (40.2%)
Age (years)	
Mean ± SD	46.66 ± 3.21
Range	37-56
Creatinine (mg/dl)	
Mean ± SD	1.03 ± 0.31
Size of the right kidney (mm)	
Mean ± SD	116.27 ± 2.33
Size of the left kidney (mm)	
Mean ± SD	115.96 ± 2.51
Stone location	
Upper ureteric	44 (47.4%)
Mid ureteric	24 (24.7%)
Lower ureteric	29 (29.9%)
Side of obstruction	
Right	51 (52.6%)
Left	46 (47.4%)
Ureteric jet frequency (jets/min)	
Non-obstructive ureter	2.89 jets/min ± 1.29
Obstructive ureter	0.70 jets/min ± 0.49

The mean cumulative stone size was 9.77 ± 2.65 mm with minimum and maximum values as 7 and 22 mm, respectively. A total of 93 (95.9%) cases had hydronephrosis; 51 (52.6%) cases had the right side of the kidney involved and 46 (47.4%) cases had the left side involved. According to the stone location, 44 (45.4%) cases had upper ureteric, 24 (24.7%) cases had mid ureteric, and 29 (29.9%) cases had lower ureteric location. The mean obstructive side jet frequency was 0.70/min ± 0.49, and the non-obstructive side jet frequency was 2.89/min ± 1.29 (P < 0.05). The mean obstructive side jet frequency was statistically the same when compared with age groups, kidney size and site, presence or absence of hydronephrosis, stone size, stone’s location in the ureter, and gender (P > 0.05).

## Discussion

Renal stone disease is one of the most common urologic diseases in the world, which demonstrates a trend toward an increase in the incidence and prevalence, especially in industrialized countries [2]. The risk factors for urolithiasis are numerous, including genetic, environmental, anatomic, and metabolic causes [2,3,7,22]. The basic mechanics posing a risk for stone occurrence are stasis of urine due to urinary tract obstruction [22].

In the past few decades, the most common imaging modalities for diagnosing urolithiasis included an x-ray or ultrasound KUB or an intravenous urography (IVU). An x-ray KUB and IVU have significant disadvantages including higher radiation exposure and considerably low sensitivity in detecting stones [8,23]. In the current era, these have largely been replaced with NCCT. A CT scan can detect the smallest and radiolucent stones; their exact location provides the Hounsfield unit of the stone and also helps with the identification of any other associated pathologies that may be quiescent. Despite the superiority of non-enhanced CT in the detection of the urinary tract, it has its demerits, including the hazard of ionizing radiation, high cost, and lack of easy availability [6,24].

**Figure 1:**
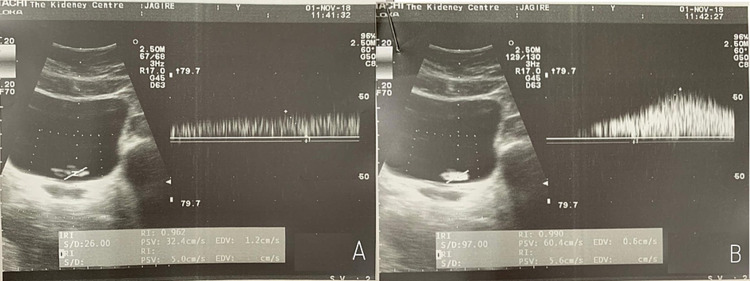
(A) and (B) Appearance of normal ureteric jet

Nonetheless, several technological advancements have made the regular ultrasound a very valuable tool that aids in the diagnosis of urinary tract pathologies, and it has remained an indispensable part of diagnostic imaging for stone disease since the 1980s [6]. The newest modality in the radiologist’s armamentarium is the Doppler ultrasound, which has completely revolutionized diagnostics in almost every field of medicine. For urology, in particular, a Doppler ultrasound helps in the visualization of the ureteric jets, which aid in the evaluation of ureteric physiology and peristaltic activity. “Ureteral jets” are defined as the flow of urine from ureters through the ureterovesical junction and into the bladder (Figures 1A, 1B) [15].

In the past, multiple studies have looked at the value ureteral jets were used for the evaluation of ureteral stone and vesicoureteral reflux disease (VUR). The results of these works indicate that that flow parameter may have an essential role in assisting with the diagnosis, by demonstrating obstruction in ureteral stones or reflux in VUR [14,25]. A few attempts have been made to assess the ureterovesical jet flow by color Doppler and duplex Doppler ultrasound [13-17]. The color Doppler and/or duplex Doppler ultrasound is proposed to be useful adjuncts to the regular grayscale method as these modalities may help us in identifying the number of intermittent urine jets emanating from the ureterovesical junction as well as the duration and peak velocity of the ureterovesical jets. These shall further improve the diagnostic accuracy of an ultrasound exam in patients with suspected ureteral stones.

Leung et al. in 2002 [16] described six different ureteral jet patterns in healthy subjects and provided information from unique demographics as well, including pregnancy, ureteral re-implantation, and VUR. However, there is no description of the pattern that may be seen in patients with ureteric stone disease. In a similar study conducted by Jandaghi et al. [18] to evaluate ureterovesical jet dynamics in the obstructed ureter, 46 patients with a diagnosis of ureteral stone underwent the grayscale ultrasound exam of both kidneys and urinary bladder. Ureterovesical jet characteristics including jet frequency, duration, and peak velocity were assessed by color Doppler and duplex Doppler studies in both obstructed and non-obstructed ureters by a radiologist. They reported the frequency of ureteric jet on the obstructed side to be 0.59 ± 0.90 jet/min in patients presenting with ureteric stones. Our study population comprised of similar patients with obstructing ureteric stones, and we observed that the mean obstructive side jet frequency was 0.70 ± 0.49 jet/min with a minimum value of 0.22 jet/min and a maximum obstructive side jet frequency of 2.89 jet/min.

However, Fields et al. in their study concluded that only ureteric jet frequency is significantly associated with ureteric obstruction, while duration and velocity of ureteric jet bear no significant correlation with ureteric obstruction [26]. Given the safety of Doppler ultrasound and the significantly observed differences in flow dynamics of obstructed versus non-obstructed ureters, the findings of this study demonstrate the utility of the Doppler ultrasound examination as a useful adjunct to grayscale ultrasound by improving the accuracy of ultrasound exam in the diagnosis of ureteral obstruction.

Another interesting study evaluated the ureterovesical jet flow as seen on Doppler ultrasound in patients with residual ureteral stone after extracorporeal shock wave lithotripsy (ESWL). They then compared these obstructed jets with the normal contralateral side. The mean peak velocity of the Doppler waveforms obtained on the residual ureteral stone was 1.71 ± 2.0 cm/sec, and the contralateral non-obstructed ureter showed a waveform of 5.60 ± 3.2 cm/sec (P < 0.05). They concluded that due to the absence of contraindications and side effects, Doppler ultrasound is a functional investigation and can contribute significantly to the diagnosis of residual ureteral stones after ESWL [19].

The results of our study indicate that the mean obstructive side jet frequency was 0.70 ± 0.49 jet/min, which in comparison to the normal contralateral ureter is significantly less. Our study has certain limitations. It is a single-center study with a limited sample size. We suggest that these useful parameters need to be looked at in much more detail prospectively and possibly at a multicenter level.

## Conclusions

From our study, we conclude that color Doppler ultrasonography can be a useful and safe modality for patients with documented ureteric stones to see if they have passed the ureteric stone after the initiation of treatment. Using the color Doppler ultrasonography, we can avoid the repeated use of imaging modalities that involve ionizing radiation and still get reasonably reliable information on whether there is still ureteric obstruction. Using ultrasonography instead of repeat NCCT not only can decrease the exposure to ionizing radiations but also can be cost-effective for the healthcare systems that are already resource-poor. It can also be used in specific populations (i.e., pregnancy) where the use of imaging modalities that involve ionizing radiation is not recommended.
